# Infra-red Thermography for High Throughput Field Phenotyping in *Solanum tuberosum*

**DOI:** 10.1371/journal.pone.0065816

**Published:** 2013-06-07

**Authors:** Ankush Prashar, Jane Yildiz, James W. McNicol, Glenn J. Bryan, Hamlyn G. Jones

**Affiliations:** 1 Cell and Molecular Sciences, The James Hutton Institute, Invergowrie, Dundee, United Kingdom; 2 Biomathematics & Statistics Scotland, The James Hutton Institute, Invergowrie, Dundee, United Kingdom; 3 Plant Science Division, University of Dundee at The James Hutton Institute, Invergowrie, Dundee, United Kingdom; 4 School of Plant Biology, University of Western Australia, Crawley, Australia; United States Department of Agriculture, United States of America

## Abstract

The rapid development of genomic technology has made high throughput genotyping widely accessible but the associated high throughput phenotyping is now the major limiting factor in genetic analysis of traits. This paper evaluates the use of thermal imaging for the high throughput field phenotyping of *Solanum tuberosum* for differences in stomatal behaviour. A large multi-replicated trial of a potato mapping population was used to investigate the consistency in genotypic rankings across different trials and across measurements made at different times of day and on different days. The results confirmed a high degree of consistency between the genotypic rankings based on relative canopy temperature on different occasions. Genotype discrimination was enhanced both through normalising data by expressing genotype temperatures as differences from image means and through the enhanced replication obtained by using overlapping images. A Monte Carlo simulation approach was used to confirm the magnitude of genotypic differences that it is possible to discriminate. The results showed a clear negative association between canopy temperature and final tuber yield for this population, when grown under ample moisture supply. We have therefore established infrared thermography as an easy, rapid and non-destructive screening method for evaluating large population trials for genetic analysis. We also envisage this approach as having great potential for evaluating plant response to stress under field conditions.

## Introduction

Most breeding effort in crop plants has focused on commercially important traits such as yield and traits directly linked to commercially important traits. For further improvements there is a need to extend the range of traits studied. Although many physiological traits are critical for plant growth and development and hence contribute to yield and to tolerance of environmental stresses, they have rarely been used in plant breeding [1]. This has largely been because of the lack of appropriate high throughput phenotyping methods to the extent that phenotypic analysis is becoming the major limiting factor in plant breeding [1], [2], [3], [4].

As accurate and elaborate phenotyping is the basis of any plant study for responses to stress, there is a need to develop robust phenotyping systems. A number of laboratory or glasshouse-based phenotyping platforms such as the Keytrack System (KeyGene, The Netherlands) and Phenofab have been developed recently (see [5], [6]). These use multiple view imaging systems including thermal sensors, together with automated plant handling under controlled environment conditions to quantify plant growth and function. However, genetic analysis and breeding for most crop species is usually carried out under natural conditions because results from glasshouse trials do not always correlate well with field behaviour [7], [8], [9], [10]. Phenotyping in field trials is therefore likely to provide better insights into crop behaviour than studies under glasshouse conditions, especially for crops such as potato that have large canopy size and show restricted growth in pots [11]. Thus, there is a strong requirement and need to establish phenotyping methods that can be used to screen large crop plant populations under natural environmental conditions.

One important physiological trait, especially in water-limited conditions, is stomatal conductance; this plays a crucial role in balancing a need to maximize photosynthesis while minimizing water loss [12]. Drought leads to stomatal closure, thus reducing water loss but with consequent reductions in photosynthesis and hence growth and yield. With increasing constraints on water availability, the efficient use of water is becoming more critical in agriculture, and manipulation of stomatal behaviour has been considered to be a likely target for improving crop water use efficiency [13], [14]. In breeding crops adapted to drought conditions it is also necessary to consider the optimal stomatal response with the need for some plasticity in stomatal behaviour so that stomata remain open when water is available but close to improve water use efficiency and survival as water deficits increase. Optimal stomatal responses depend on the probability of future rainfall [12], [15], [16]. Thermal imaging has been shown to be a particularly sensitive method for the study of stomatal conductance [17] and is especially useful for screening mutant populations because it averages large areas of canopy, and is hence much more rapid and provides greater replication than the use of porometry [18], [19], [20].

A number of previous studies have demonstrated relationships between stomatal conductance and the commercially critical trait of yield. For example, work on rice has shown that genotypic differences in grain yield in rice were closely related to crop growth rate, which in turn was closely related to higher stomatal conductance during the two–week period preceding full heading [21], [22], [23]. Similar results have been found for wheat and cotton [24], [25], [26], [27], [28]. These latter results have also shown a relationship between stomatal conductance and yield that is independent of photosynthetic rate [28], [29].

These reports suggest that there is therefore good reason to focus on screening for stomatal conductance as a means for improving yield, while the importance of stomatal conductance in controlling water loss suggests that it will be particularly important under water-limited conditions. The work described in this paper aims to explore the reproducibility and sensitivity of canopy-scale thermal imaging (by infra-red thermography (IRT)) as a tool for assessing the genetic variation in stomatal conductance in a diploid potato mapping population. We report the first extensively replicated trials of large scale thermal phenotypic screening under field conditions, with strong evidence for good reproducibility at different times of the day and on different days. We also evaluate and compare methods for processing, assessment and use of images and the requisite statistical methods to eliminate the effect of varying weather conditions over time. We further investigate whether there is any relationship between the observed canopy temperature and yield.

## Materials and Methods

**Figure 1:**
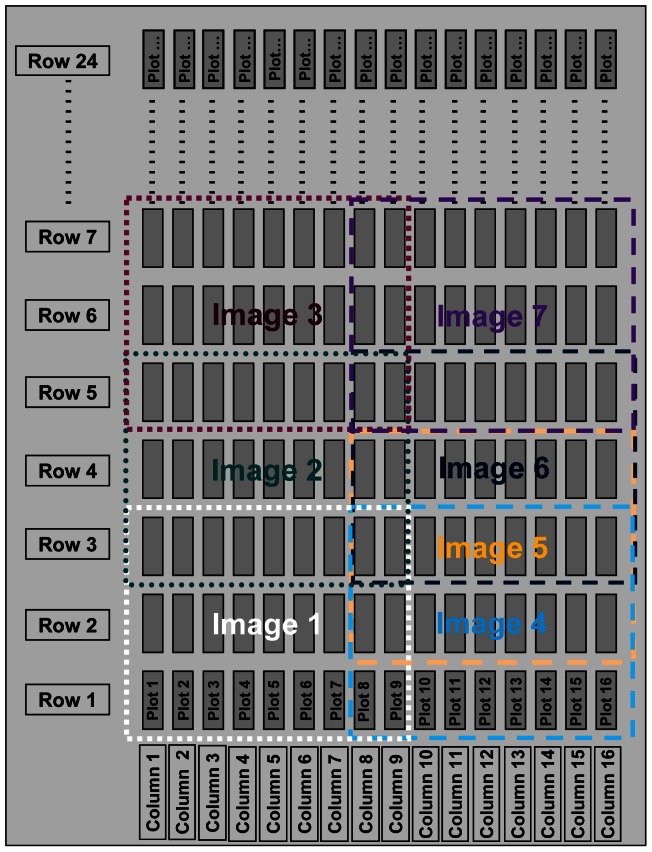
An example of field trial layout showing the imaging strategy. The sequence of images is illustrated showing the overlap between images with many plots having repeated measurements. Each plot in the plan contains a single genotype plot (2 or 5 plants). So a single trial with 192 genotypes replicated twice has 24 rows of 16 plots.

The work was carried out on a biparental diploid potato population (06H1), derived from a cross between two hybrid clones (99FT1b5 and HB171(13)) each of which are clones derived by crossing *Solanum tuberosum* group Phureja and *Solanum tuberosum* group tuberosum (manuscript in preparation). In this study 188 clones from the population together with the two parents and two commercial varieties (Record and Cara) were planted in the field as a replicated trial using an alpha design [30]. Field trials were conducted in 2011 at Balruddery farm, The James Hutton Institute (Latitude 56.48°N, Longitude 3.13°W). Field trials were established with separate independent plots each representing a harvest to take place at five different time points (stolon initiation to tuber initiation) during the life cycle of potato (described as Trial 1, Trial 2, Trial 3, Trial 4 and Trial 5). Each of these trials comprised the selected 192 clones in the form of two-plant plots replicated twice except for trial 5 which had five plant plots replicated twice and was used to assess the final yield. The trials were planted in 16 plot rows and the genotypes were randomized according to an alpha design with small block size of 8 (as an example, Trial 1 is shown in Figure 1).

### Acquisition of infrared images

**Figure 2:**
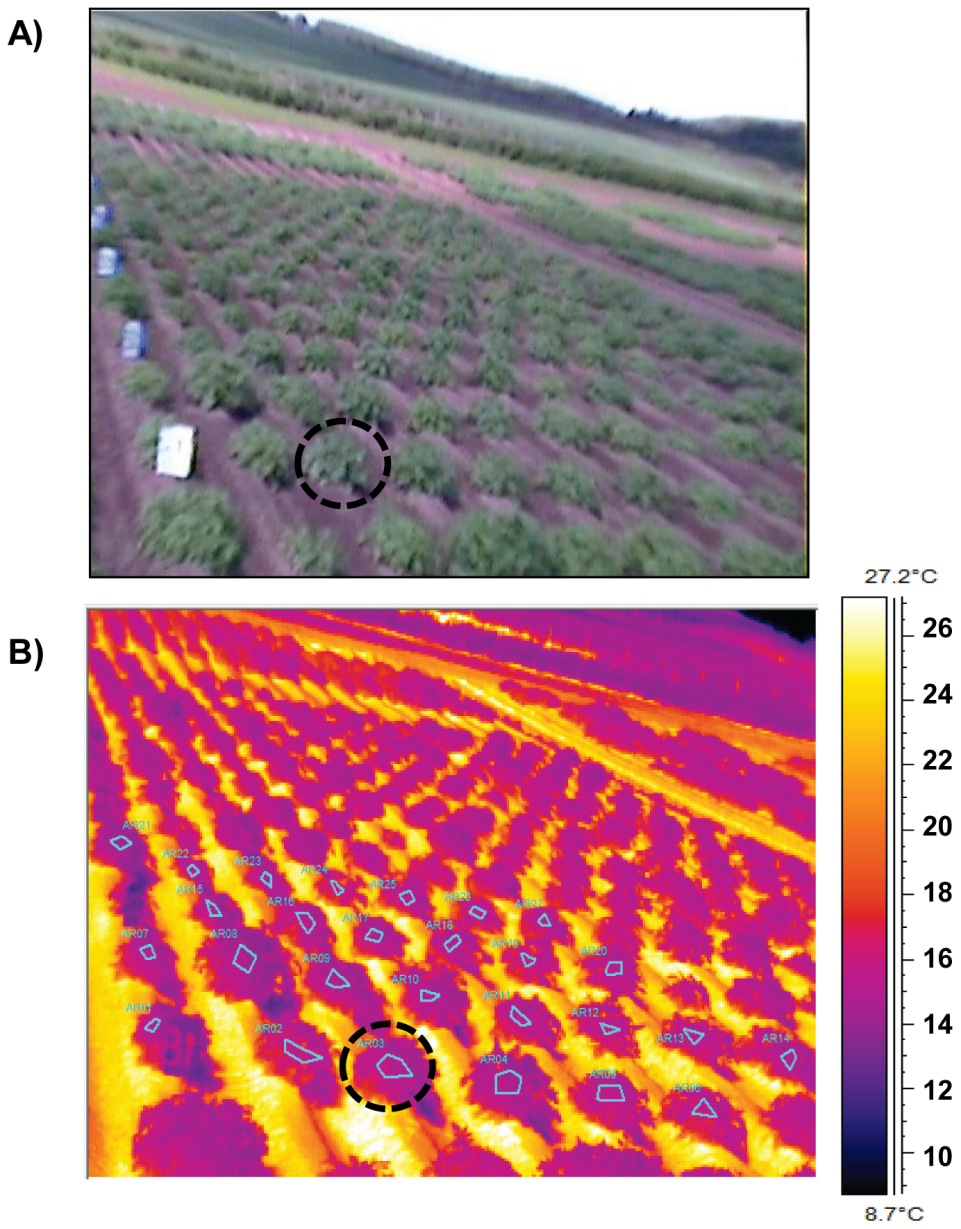
Visual (RGB) image and corresponding thermal image of part of the potato field trial. **A**): Visual image of the randomised potato field trial showing markers to aid localisation in the thermal image. The marked circle indicates one of the two-plant plots. **B**) Corresponding thermal image; this shows the selected area of canopy for each plot isolated in the ThermaCAM Researcher Pro software.

Thermal images were obtained using a ThermaCAM P25 infrared camera (FLIR systems, USA) that operates in the spectral range of 7.5–13 µm and has a focal plane array (FPA) uncooled microbolometer detector with a spatial resolution of 320×240 pixels. Images were taken from a fork-lift at about 8 m height and covered up to 9 plots horizontally and 3–4 rows (Figure 1 and Figure 2). Thermal images were taken at 3 developmental stages (28 June and 5 and 12 July: referred as day 1, day 2 and day 3 in the rest of paper) with images on day 1 for all the 5 trials, day 2 for trials 2, 3, 4 and 5 and on day 3 for trials 3, 4 and 5. Images were taken at an angle to maximise the number of individual plots in any image [20]. Data acquisition took up to 6 hours on day1 (between 9:30 am and 3:00 pm) as imaging took nearly 0.75 h for each trial and imaging on the consequent days took slightly less with similar time between trials (Table S1). The camera parameters such as reflected, atmospheric and optics temperatures (20°C) and relative humidity (40%) were kept constant through the whole experiment. The absolute error this causes did not affect our analytical approach which depends on relative values only.

### Thermal Image Analysis

Thermal images were processed using ThermaCAM Researcher Pro 2.8 SR-1 software (FLIR systems). To estimate the canopy temperature of each plot (genotype), the acquired images were loaded into the ThermaCAM software and typical areas of canopy for each plot were selected by drawing polygons, avoiding edges and patches of bare ground. Plot temperature was estimated by the mean temperature of the enclosed pixels (Figure 2). As a quality control measure to ensure that no ground-pixels (as opposed to leaf-pixels) had been included, the histogram of all the individual pixel temperatures in each polygon was used as a visual check to ensure that no temperature outliers had been included in the sampled area.

### Data analysis and Statistics

Plot canopy temperatures estimated from the thermal images were analysed using GenStat (ver-14.1.0.5943; VSN international Ltd) and R statistical software (ver 2.14.0; The R Foundation for Statistical computing). Two methods of normalizing the plot temperatures were considered, regression analysis (REML) and a simple differencing approach. The significance of differences among genotypes was determined using analysis of variance. REML analysis was performed in GenStat using Trial, Genotypes and Picture temperature as fixed effects with genotypic means and picture temperature means predicted by averaging across trials.

### Measurement of yield, height and maturity

In order to find the relationship between the canopy temperature and yield of the genotypes, tuber yield was assessed at physiological maturity. Yield is represented as harvested yield from five plants from Trial 5. To explore the relationship between thermal data and yield before physiological maturity, tuber yield was also measured for Trial 4. Maturity was assessed quantitatively for trial 5 on a scale of 1–9, where 9 is less mature and 1 is most mature.

## Results

### Acquisition and normalisation of Images

**Figure 3:**
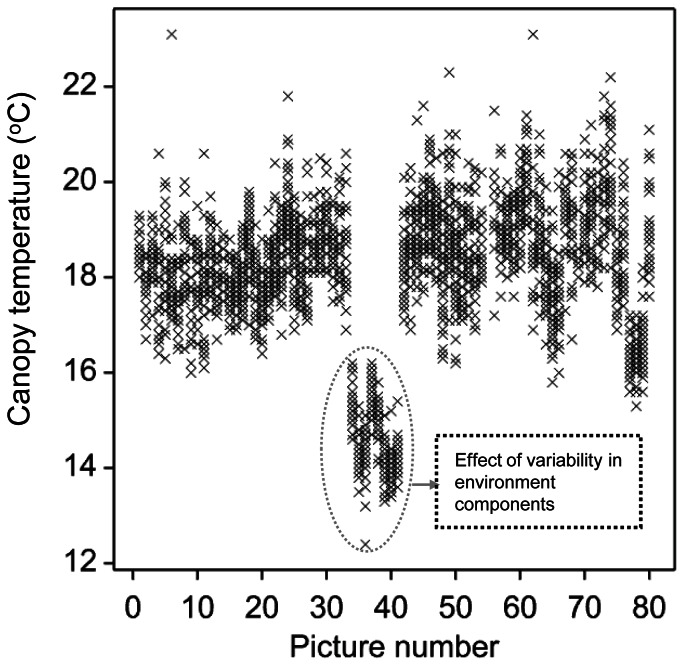
Canopy temperature (°C) variation within images and between successive images. Dot plot covering measurements on three trials on a single day showing the substantial temporal variation, with the major reduction in temperatures between images 34 and 42 resulting from variability in environmental components. Each cross represents the temperature of a single plot in one image.

The procedure for acquiring the thermal images involved deliberate but non-systematic overlapping and therefore most plots appeared in two or more images (Figure 1). There are two main consequences of image overlapping, firstly that the increased replication reduces the standard error of the genotype means. Secondly it provides scope for better normalisation of each image for changing environmental conditions such as increasing or decreasing cloud cover. Figure 3 shows image-to-image variation in the raw individual image plot temperatures (referred to as IPTs from now on) for 80 consecutive images covering 3 trials taken on a single day. The between-image variation was caused by environmental changes such as cloud cover (and irradiance), wind speed, temperature and humidity [17], [20]. Therefore the plot temperatures in each image were normalised by subtracting the mean temperature of all the plots in that image, giving the individual-image normalised plot temperature (IINPT). The normalised plot temperature (NPT) for plots occurring in more than one image was estimated as the mean of the IINPTs (for individual plots across different images). The NPTs were then used in all subsequent statistical analyses. Genotypic means or normalised genotype temperatures were obtained by averaging across replicates for each trial. A more sophisticated approach using REML to estimate genotype means, adjusted for Trial, Image, time and replication generated similar genotype estimates. Comparison of these two approaches showed that the estimated genotype means were similar and Pearson correlation coefficients between the estimated genotype means were >0.95 for all trials, with no evidence for systematic bias (data not shown). Therefore it was decided to proceed with the more direct image-differencing approach rather than technically more complex REML.

**Figure 4:**
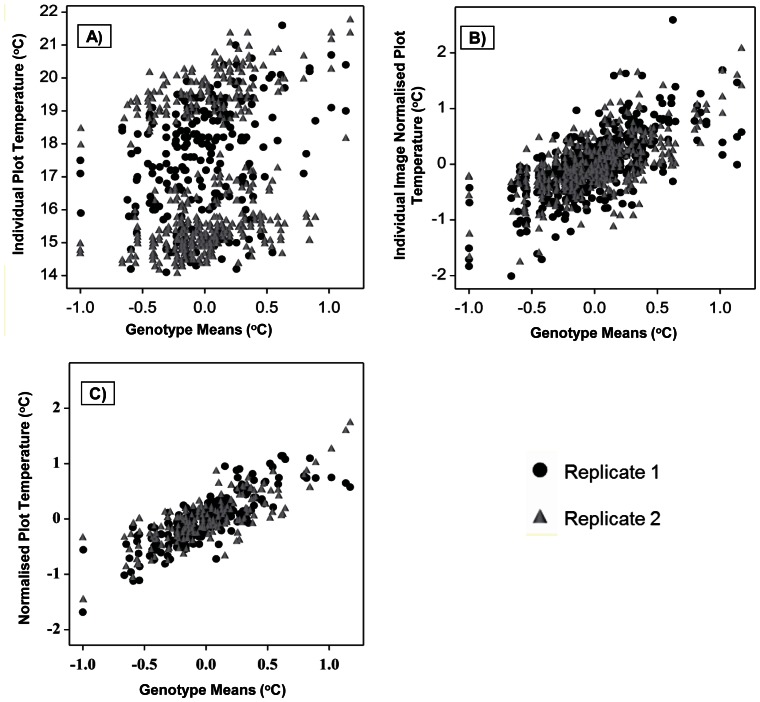
Effect of replication and normalisation. Illustration of the benefits of image overlaps and of normalisation for calculating the genotype means during thermal image analyses (using the example of Trial 4_Day1 data). The two replicates are indicated using different symbols. **A**) Relationship between individual plot temperature (IPT) on y-axis and Genotype Means. **B**) Relationship between the “individual-image normalised plot temperature” (IINPT) and Genotype Means. **C**) Relationship between Normalised Plot Temperature (NPT) and Genotype Means. Note: Residual errors as a measure of the difference.

The overlap strategy and the normalisation technique used above assist in analyzing data with more precision and reduction in standard error of the genotype means. This is shown in Figure 4, where comparisons between different scatter plots demonstrate that the degree of variability decreases with increased replication and use of normalisation. The comparison in Figure 4 is made with Genotype means on the x-axis, with the y-axis representing individual plot temperatures (IPT) in Figure 4(A), the individual-image normalised plot temperatures (IINPT) in 4(B) and normalised plot temperature in 4(C). Figures 4 (A) and 4 (B) shows the effect of normalisation, whereas comparison between 4 (B) and 4(C) shows the benefit of increased replication as a result of overlap between images, while comparison of Figures 4 (A) and 4 (C) illustrates the benefit of both normalisation and replication.

### Reproducibility of thermal data

Thermal images were taken on the 192 genotypes on three different days, during the growing season of the potato plants between stolon initiation and tuber initiation. Data for canopy temperature on each trial measured on different days were analysed separately as 12 independent sets of thermal imaging data (with replication in each trial). This allows us to assess the consistency and reproducibility of the thermal data (between trials/time of the day and between days).

#### Reproducibility at different physiological stages

**Figure 5:**
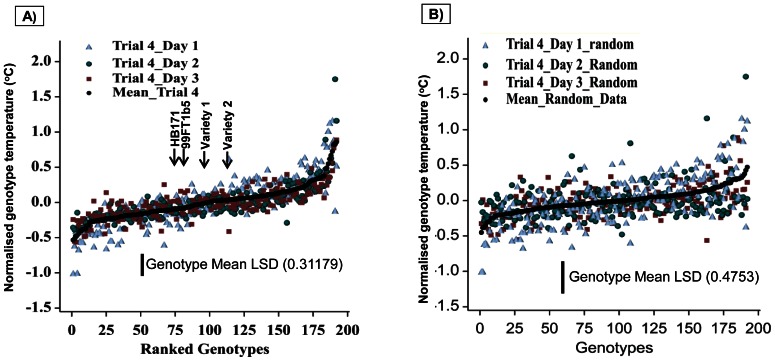
Variation of the normalised temperature for each genotype ranked by mean temperature. **A**) Graphical representation of the consistency of the rankings of the different genotypes across three measurement dates for Trial 4. The genotypes are ranked along the x-axis according to the mean of all the replicate normalised genotype temperatures (°C) over three days. The X-axis also indicates the positions for the two parents (HB171, 99FT1b5) and two commercial varieties (Var1 = Record and Var2 = Cara). **B**) The same data as (A) but with data randomly assigned to ‘genotypes’ independently on each day, indicating the range of genotype means that could have arisen by chance. This confirms that the substantial genotypic differences in mean normalised temperature shown in (A) are not due to chance. LSD values presented at 95% level in all graphs.

Analysis of variance of NPT for trial 4 (Table 1) shows that the interaction between days and genotypes and also variation due to day of imaging is not significant and thus provides evidence for substantial consistency in the genotypic ranking of canopy temperature over the three days (covering the period between stolon initiation and tuber initiation). These results are plotted in Figure 5(A) and show substantial differences in mean normalised temperatures between extreme genotypes (ANOVA on data for means for two replicates for trial 4 on different days: between-genotype MS = 0.1736, df = 191 and within-genotype MS = 0.0378, df = 384, p<0.001 with genotypes explaining 54.5% variation). As a test of whether these results could have arisen by chance, a repeated randomization approach (Monte Carlo analysis) was applied to the data. In this approach the allocation to genotype was repeatedly randomised separately for each day. This allowed calculation of an apparent LSD that could have arisen by chance. The results of one simulation are plotted in Figure 5(B) where the ‘simulated genotype’ range was approximately twice the apparent LSD. The result from analysis of variance on such randomized data show a non-significant main affect (between-genotype MS = 0.074 and within-genotype MS = 0.087, p = 0.91). Similar results were obtained for all trials (data not shown).

**Table 1:** Analysis of variation testing the consistency and significance between different trial days and genotype and day interaction for the Trial 4 thermal data.

Source	df	MS	F ratio	p-value
Genotype	191	0.33353	4.65	<0.001
Days	2	0.08795	1.22	0.295
Days * Genotype	382	0.07177	0.86	0.946
Error	572	0.08354		

#### Within day reproducibility

**Figure 6:**
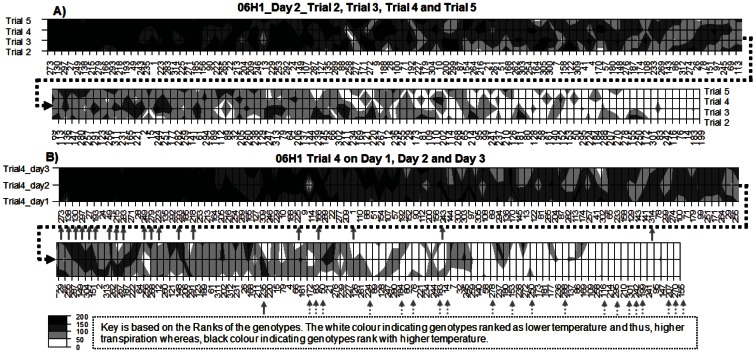
Consistency in genotype ranking at different times and at different physiological ages. **A**) Contour plots illustrating the consistency in genotype ranking for normalised genotype temperature (genotype means) between different trials on the same day but measured at different times (Trial 2, 3, 4 and 5 on Day 2, imaged between 9:00 am to 12:30 pm, refer to Table S1). **B**) Contour plots illustrating the consistency in genotype ranking for normalised genotype temperature between different days for one trial (Trial 4 on day1 (28/06), day2 (05/07) and day3 (12/07/2011)). The arrows in (B) correspond to the positions of the 20 genotypes each rank extreme in (A) with dotted arrows indicating the coolest and solid arrows the hottest. Data are expressed as genotype rankings on each occasion (x-axis indicating genotype numbers), with black areas indicating genotypes ranked among the hottest on any occasion and white areas indicating genotypes ranked among the coolest.

**Figure 7:**
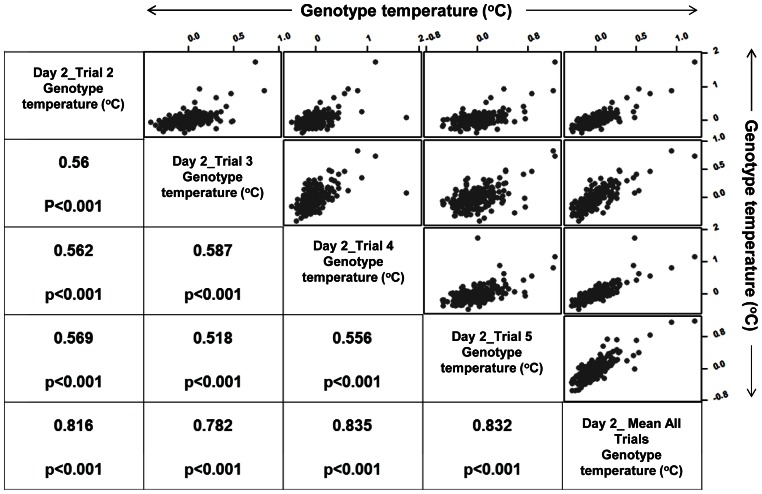
Correlation between trials. A representative extract from the full matrix showing both the correlations between normalised genotype temperatures (represented as °C) for different trials and measurement days and the associated correlation coefficients.

The results on normalised plot temperature data also show good reproducibility in genotype rankings between trials measured at different times of day (Figure 6(A) and Table S2). Rank correlations for the 20 genotypes from each extreme selected for high and low temperature based on mean ranks on these day 2 trials exhibit a high consistency in normalised canopy temperature (Spearman's rho range = 0.802 to 0.918 with p<0.001 for the 4 Trials). Similarly, a comparison of rankings at different physiological stages (Figure 6(B)) also showed substantial consistency, with the data for the 20 extreme genotypes (Spearman's rho = 0.943, p<0.001, based on 20 genotypes from each extreme selected on mean ranks of day 2 Trials data). Figure 7 and Figure S1 shows the correlations between normalised temperatures on different trials on day2. In each case the data are highly positively correlated. The correlations between Day 1_Mean, day 2_Mean and Day 3_Mean, where Mean is the average of different trials on that day, are higher than those for the individual trials, suggesting that repeated measures of thermal data creates higher replication, thus minimizing the variation.

### Canopy temperature and harvest traits

**Figure 8:**
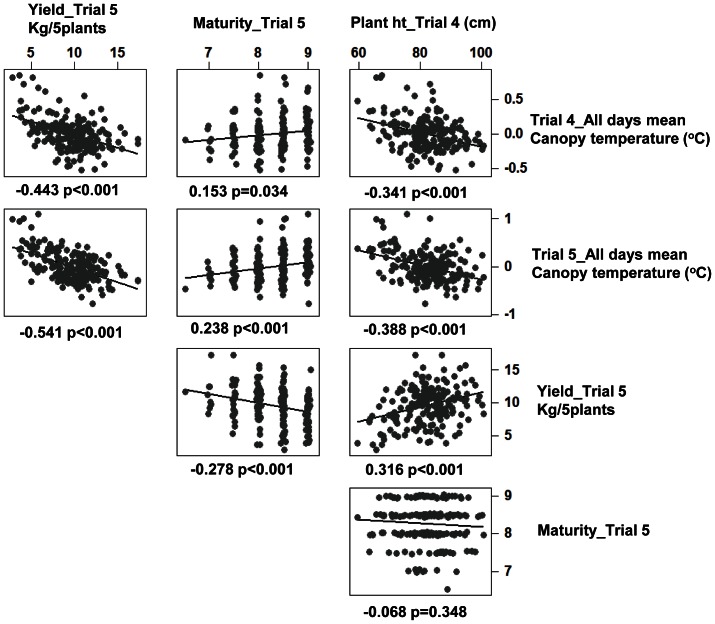
Genotype temperatures and harvest traits. Relationship between normalised genotype temperatures (mean of three days) for Trials 4 and 5 and harvest traits (Yield, Plant height and Maturity) represented using regression lines in the plots. The numerical figures displayed provide the correlation coefficients and the *p* values for the plotted traits.

The relationships between canopy temperature for different trials and days and growth traits (yield, maturity and plant height) are presented in Figure 8 and Figure S2: this shows a consistent negative relationship between canopy temperature and yield (r = 0.541, p<0.001, Trial 5_Mean and Yield). A similar relationship was found between canopy temperature and yield in trial 4 (r = 0.531, p<0.001, data not shown). Final yield was related to yield at trial 4 (r^2^ = 55%, p<0.001). The yield data for trial 4 were collected roughly 3 months after planting (at the stage of tuber bulking) whereas the yield data for trial 5 were collected at maturity (around 2 months after trial 4 harvest). Previous studies show that evaporation is the main determinant of leaf temperature and there is a direct relationship between leaf temperature, transpiration rate and stomatal conductance [20], [31]. These results imply that the higher yielding phenotypes have higher transpiration.

Stepwise multiple regression using a forward selection approach was used to determine the importance of canopy temperature, plant height and maturity as predictors of yield. Similar results were obtained using best subsets regression (data not shown). The majority of variation in yield was associated with variation in canopy temperature (r^2^ = 29.2%) and when interacting with maturity it explains nearly 31.6% of the variation and on further addition of plant height, the amount of variation explained is 33%. Thus the results show that stomatal conductance or canopy temperature as measured in these trials play a significant role in determining yield, though there are possibly other morphological and physiological traits that determine yield in interaction with this trait in potato.

Canopy temperature shows weak correlations with plant height (ranging from r = −0.215, p<0.003 to −0.396, p<0.001) and maturity (ranging from non-sig to r = 0.238, p = 0.001) where maturity is scored on 1 to 9 basis with 9 considered as less mature, thus suggesting that genotypes with higher canopy temperature mature later.

## Discussion

### Normalisation of data

The surface temperature of the plant canopy depends on both biological and environmental factors. As our experimental design enabled canopy temperatures to be measured simultaneously for large numbers of genotypes, the normalisation technique employed minimises environmentally-caused temperature variation, leaving primarily the genetic differences. The practice of overlapping different images increases replication, thus allowing more precise estimation of temperature differences and thus reducing the standard error of the plot/genotypic means (Figure 4). Increased replication helps increase the significance of between genotypes; this should help increase the power to locate QTLs for this trait [32].

### Reproducibility of data

Jones et al. (2009) demonstrated that clear genotypic variation can be detected by using normalisation techniques even where substantial variation in soil moisture exists and have given examples of the application of infra-red thermography to phenotyping in the field [20]. In this paper we extended this approach to a large scale multi-trial experiment to investigate the power of normalisation techniques to improve the consistency and sensitivity of canopy temperature data. The results confirm that “between-genotype” variation can be consistently detected among a population of potato lines when using appropriately normalised thermal data (Figures 5 and 6). The reproducibility of the data after normalisation was not affected by either the time of measurement during the day or the physiological stage of the plant.

Although the replication of genotypes within a trial sets the limits to the power of a trial, the errors involved in thermal imaging, including those resulting from environmental variation between images, further degrade the power of any such trial. We have shown that this further error can be minimised by enhanced replication of genotype data either by using ‘image overlap’ or by increasing the number of images taken. The use of normalised temperatures instead of the raw temperatures is crucial in eliminating the main effect of environmental differences (e.g. air temperature, humidity or irradiance) during the day or between days (Figure 4).

The matrix of correlation plots in Figure 7 and Figure S1 illustrates the high levels of consistency between results for different trials and on different days. The correlations are highest between the daily means, indicating the advantage of enhanced replication using the independent trials.

### Relationship of temperature to canopy height

Negative associations of temperature with plant height, similar to those observed in this paper have been observed in wheat, where it was hypothesized that the lower temperature for taller crops was related to a higher boundary layer conductance with taller crops [33], [34]. It remains unclear what underlies the lower temperatures observed with taller crops. Although this may simply result from a true correlation between height and stomatal conductance; as we do not have stomatal conductance data we cannot rule out the possibility of an aerodynamic effect contributing to the difference and reducing the power of the thermal measurements to discriminate conductance [35]. Despite the fact that maturity for this cross has not been studied extensively (due to it being a uniformly late maturing population) the results in this paper indicate that the plants which are less mature have lower yields: this late maturity is also associated with higher canopy temperature. Thus, this suggests that under our optimum water conditions the plants with higher stomatal conductance have short life cycle or mature early.

### Potential for Potato Research

Infrared thermography (IRT) is a powerful tool for studying plant responses to environmental stress and for screening plants for differences in stomatal conductance, as changes in canopy temperature are driven by differences in stomatal conductance, which itself is particularly sensitive to water deficit stress [17]. IRT has many advantages over other methods such as the use of a porometer for the phenotyping of stomatal behaviour as it is both more rapid and non-destructive, and it averages the whole canopy instead of individual leaves. It has therefore been used extensively for identifying and monitoring plant stress and as an aid in irrigation scheduling [31], [36]. In this study we have shown that even with ample water supply there is substantial variation in canopy temperature between different potato genotypes. The highly heterozygous population evaluated here showed temperatures varying by ∼2.1°C, even under cool conditions with a maximum environmental temperature of 18°C. A substantial portion of the temperature variation observed was genotypic as there was a good consistency between genotypic rankings for different days and for different trials. The potato population used in this experiment has shown significant variation among genotypes for canopy temperature and is being used further to associate with a large set of Single Nucleotide Polymorphisms (SNPs) for mapping regions which control canopy temperature/stomatal conductance. Future work will investigate the behaviour of these genotypes under irrigated and water stress conditions to identify genotypes which are stress tolerant and also which are transpiring more efficiently {by combining IRT data with Carbon Isotope signatures (delta^13^C)}. The combination of a mapping approach and the study of genotypic responses to water availability will allow one to quantify the genetic differences in stomatal behaviour. Potentially this will help in breeding genotypes which can close stomata and conserve water under drought conditions but take advantage of any available water. The results described here have identified the extreme genotypes in terms of constitutive stomatal behaviour under well-watered conditions, while the new trials will concentrate on responsiveness to drought allowing mapping of relevant QTLs.

### Potential as an indirect trait

The present results also provide evidence for significant negative correlations between yield and canopy temperature as would be expected if increased stomatal opening led to enhanced yield. For example for the trial 5 mean thermal data the correlation with yield was (−0.541, p<0.001) while similar correlations were found for trial 4 data as well.

It has been shown in rice [22] that canopy diffusive conductance estimated by remote sensing can be an effective criterion for the selection of high-yielding rice genotypes, while cooler canopies in wheat have been consistently positively associated with grain yield in other cases [37]. This association between grain yield and lower canopy temperatures has been related to a greater aerial biomass and higher stomatal conductance and photosynthesis [25], [38]. Similarly canopy temperature depression and stomatal conductance have been positively correlated with the increased linear progression in grain yield in spring wheat over six years [25]. In the present study the highest association between temperature and yield was obtained for temperature measurements at earlier stages of development. Further work is being carried out to explore the linkage between stolon and tuber initiation and thermal data.

The present results suggest a negative correlation between canopy temperature and plant maturity, thus the plants which have high transpiration rate have shorter life cycle in this population. However, as this cross is between the hybrids of *S. tuberosum* group phureja and *S. tuberosum* group tuberosum with a limited range of maturity scores (6–9), this result is only tentative and needs further evaluation.

We conclude that the use of IRT is an easy, rapid and non-destructive method of screening for stomatal behaviour and can be used for evaluating large population trials for genetic analysis. We have also demonstrated a link between high stomatal conductance and high yielding genotypes under favourable growing conditions which suggest that thermal imaging may provide a useful screen in programmes aiming to increase yield under ample water availability and consider that it may have similar potential under stress conditions.

## Supporting Information

Table S1 Half-hourly means of meteorological data over the period of thermal measurements obtained at a location around 800 m from the trial site; start times for imaging of each trial are indicated.
(DOCX)

Table S2 Analysis of Variance testing the consistency and significance between different trials on a day.
(DOCX)

Figure S1 **Matrix plot showing both the correlations between normalised genotype temperatures (°C) for different trials on different measurement days and the associated correlation coefficients.**
(TIF)

Figure S2 **Relationship between normalised genotype temperatures (°C) for Trial 4 and 5 on 3 days of infra-red imaging and harvest traits (Yield, Plant height and Maturity) represented using regression lines in the plots.** The numerical figures on left side of the graphical representation provide the correlation coefficients and the p values for the plotted traits for each plot respectively. Relationships between harvest traits and canopy temperature are also shown for the data on “Average of All traits” on day 1, day 2 and day 3 and also for data on trial 4 and trial 5 averaged over three days of imaging. Units: Yield in kg/5plants, Plant height in cm.
(TIF)
