# Supplementary material for: Infra-red Thermography for High Throughput Field Phenotyping in Solanum tuberosum
Source: PLoS One. 2013 Jun 7;8(6):e65816. doi: 10.1371/journal.pone.0065816 (PMC3676367; doi:10.1371/journal.pone.0065816)
Supplement: Table S1 — Half-hourly means of meteorological data over the period of thermal measurements obtained at a location around 800 m from the trial site; start times for imaging of each trial are indicated. (DOCX) [file pone.0065816.s001.docx]

| **Date** | **Time** | **Temperature (^o^C)** | **RH (%)** | **Solar_Rad W m^-2^** | **~Thermal Imaging time** | **Trial** |
| --- | --- | --- | --- | --- | --- | --- |
| 28/06/11 | 09:00 | 13.1 | 66 | 535 |  |  |
| 28/06/11 | 09:30 | 13.9 | 59 | 603 | 09:40 | Trial 1 |
| 28/06/11 | 10:00 | 14.5 | 60 | 667 |  |  |
| 28/06/11 | 10:30 | 15.2 | 57 | 720 | 10:30 | Trial 2 |
| 28/06/11 | 11:00 | 15.5 | 52 | 772 |  |  |
| 28/06/11 | 11:30 | 16.1 | 58 | 778 | 11:30 | Trial 3 |
| 28/06/11 | 12:00 | 16.5 | 53 | 862 |  |  |
| 28/06/11 | 12:30 | 17.0 | 54 | 696 |  |  |
| 28/06/11 | 13:00 | 17.3 | 55 | 864 | 13:10 | Trial 4 |
| 28/06/11 | 13:30 | 17.4 | 54 | 723 |  |  |
| 28/06/11 | 14:00 | 18.2 | 51 | 911 | 14:00 | Trial 5 |
| 28/06/11 | 14:30 | 18.5 | 49 | 989 |  |  |
| 28/06/11 | 15:00 | 17.5 | 52 | 582 |  |  |
|  |  |  |  |  |  |  |
| 05/07/11 | 09:00 | 14.3 | 91 | 146 | 09:15 | Trial 2 |
| 05/07/11 | 09:30 | 14.3 | 91 | 125 |  |  |
| 05/07/11 | 10:00 | 14.7 | 90 | 206 | 10:15 | Trial 3 |
| 05/07/11 | 10:30 | 14.9 | 89 | 222 |  |  |
| 05/07/11 | 11:00 | 15.8 | 85 | 347 | 11:10 | Trial 4 |
| 05/07/11 | 11:30 | 16.1 | 85 | 322 |  |  |
| 05/07/11 | 12:00 | 16.6 | 83 | 420 | 12:00 | Trial 5 |
| 05/07/11 | 12:30 | 16.1 | 85 | 336 |  |  |
| 05/07/11 | 13:00 | 15.9 | 86 | 272 |  |  |
|  |  |  |  |  |  |  |
| 12/07/11 | 09:00 | 12.5 | 75 | 192 |  |  |
| 12/07/11 | 09:30 | 12.7 | 75 | 215 | 09:30 | Trial 3 |
| 12/07/11 | 10:00 | 13.3 | 73 | 287 |  |  |
| 12/07/11 | 10:30 | 13.5 | 72 | 245 | 10:20 | Trial 4 |
| 12/07/11 | 11:00 | 13.7 | 71 | 314 | 11:10 | Trial 5 |
| 12/07/11 | 11:30 | 13.5 | 72 | 241 |  |  |
| 12/07/11 | 12:00 | 13.8 | 73 | 314 |  |  |

**Table S1:** Half-hourly means of meteorological data over the period of thermal measurements obtained at a location around 800 m from the trial site; start times for imaging of each trial are indicated
