# Supplementary material for: Infra-red Thermography for High Throughput Field Phenotyping in Solanum tuberosum
Source: PLoS One. 2013 Jun 7;8(6):e65816. doi: 10.1371/journal.pone.0065816 (PMC3676367; doi:10.1371/journal.pone.0065816)
Supplement: Table S2 — Analysis of Variance testing the consistency and significance between different trials on a day. (DOCX) [file pone.0065816.s002.docx]

**Table S2:** Analysis of Variance testing the consistency and significance between different trials on a day.

| 1. **Day 1 All trials** | | | | |
| --- | --- | --- | --- | --- |
| **Source** | **df** | **MS** | **F-ratio** | **p-value** |
| Trial | 4 | 0.0235 | 0.084 | 0.98 |
| Genotype | 191 | 1.0385 | 3.895 | <0.001 |
| Trial*Genotype | 761 | 0.2691 | 1.009 | 0.45 |
| Error | 934 | 0.2666 |  |  |
|  |  |  |  |  |
| 1. **Day 2 All Trials** | | | | |
| **Source** | **df** | **MS** | **F-ratio** | **p-value** |
| Trial | 3 | 0.0284 | 0.653 | 0.58 |
| Genotype | 191 | 0.2405 | 5.529 | <0.001 |
| Trial*Genotype | 573 | 0.0424 | 0.975 | 0.62 |
| Error | 564 | 0.0435 |  |  |
|  |  |  |  |  |
| 1. **Day 3 All Trials** | | | | |
| **Source** | **df** | **MS** | **F-ratio** | **p-value** |
| Trial | 2 | 0.0192 | 0.386 | 0.68 |
| Genotype | 191 | 0.1734 | 3.488 | <0.001 |
| Trial*Genotype | 382 | 0.0512 | 1.030 | 0.37 |
| Error | 572 | 0.0497 |  |  |
