# Supplementary figures and images for: Infra-red Thermography for High Throughput Field Phenotyping in Solanum tuberosum
Source: PLoS One. 2013 Jun 7;8(6):e65816. doi: 10.1371/journal.pone.0065816 (PMC3676367; doi:10.1371/journal.pone.0065816)

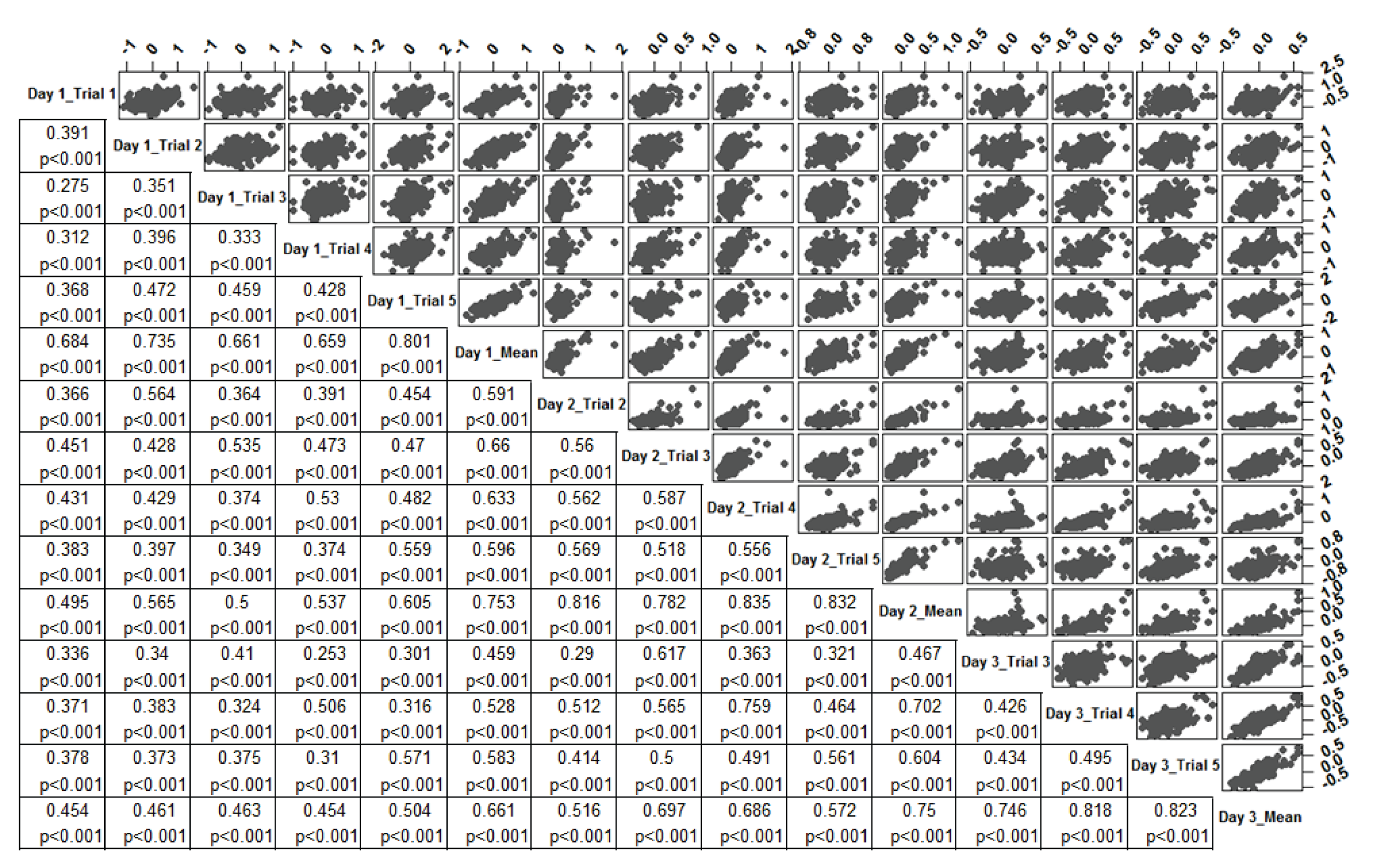

Supplement: Figure S1 — Matrix plot showing both the correlations between normalised genotype temperatures (°C) for different trials on different measurement days and the associated correlation coefficients. (TIF) [file pone.0065816.s003.tif]

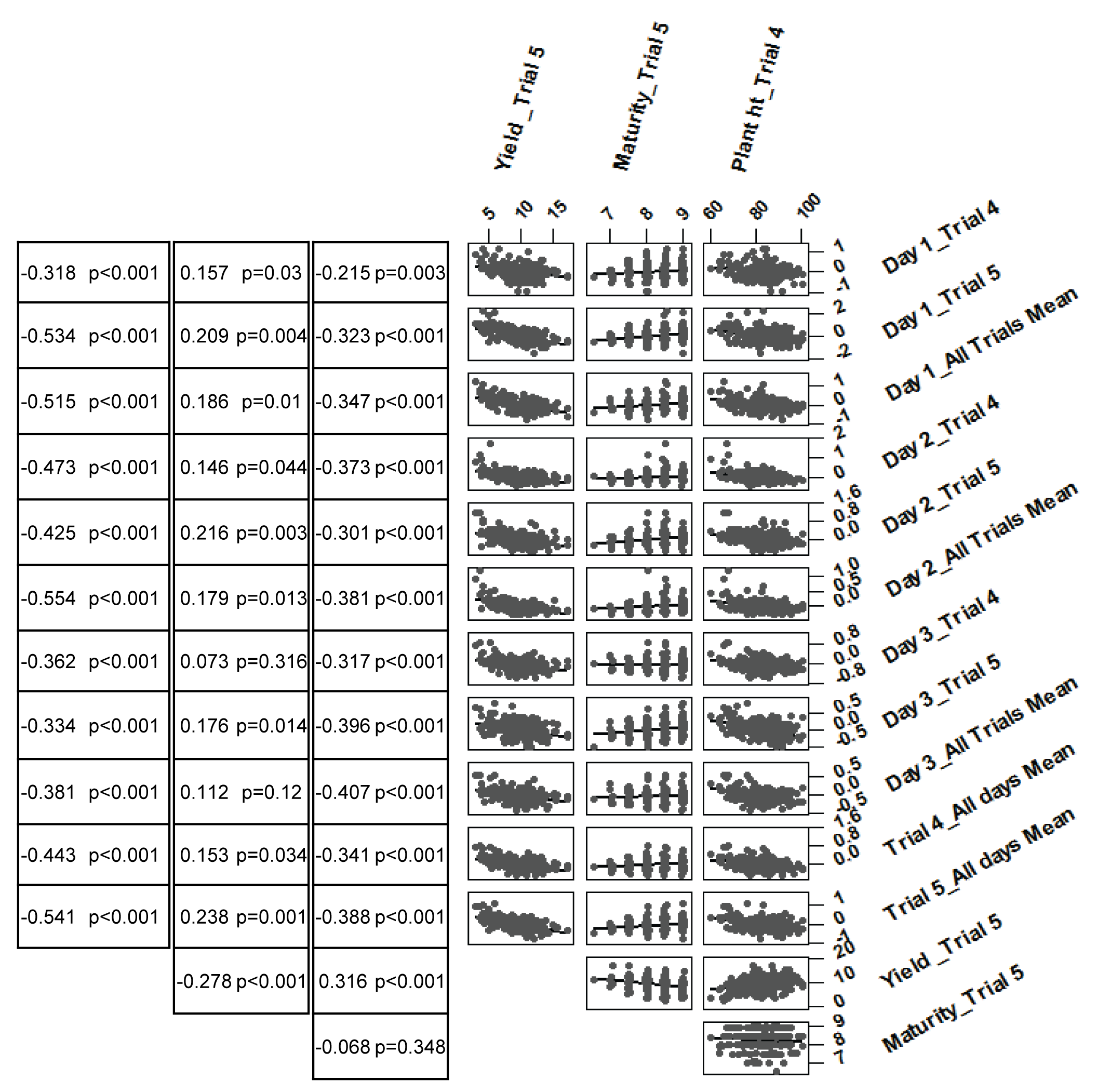

Supplement: Figure S2 — Relationship between normalised genotype temperatures (°C) for Trial 4 and 5 on 3 days of infra-red imaging and harvest traits (Yield, Plant height and Maturity) represented using regression lines in the plots. The numerical figures on left side of the graphical representation provide the correlation coefficients and the p values for the plotted traits for each plot respectively. Relationships between harvest traits and canopy temperature are also shown for the data on “Average of All traits” on day 1, day 2 and day 3 and also for data on trial 4 and trial 5 averaged over three days of imaging. Units: Yield in kg/5plants, Plant height in cm. (TIF) [file pone.0065816.s004.tif]
